# Extracorporeal membrane oxygenation for life-threatening measles in children: a case series

**DOI:** 10.1007/s00431-026-07467-4

**Published:** 2026-10-01

**Authors:** Salmas Watad, Adi Avniel Aran, Asaf Mandel, Yshia Langer, Ezra Weinblatt, Isaac Manaster, Natalie Barsoum, Sergei Elber Dorozko, Dina Averbuch, Oren Gordon, Uri Pollak

**Affiliations:** 1 Section of Pediatric Critical Care, Hadassah University Medical Center, Ein Kerem, POB 12000, Jerusalem, 9112001 Israel; 2 Faculty of Medicine, The Hebrew University of Jerusalem, Jerusalem, Israel; 3 Pediatric Infectious Diseases Unit, Hadassah University Medical Center, Jerusalem, Israel

**Keywords:** Coinfection, Extracorporeal membrane oxygenation, Intensive care unit, Pediatric, Measles, Shock, Septic

## Abstract

Measles is resurgent worldwide, and although vaccine-preventable, severe disease can progress to cardiorespiratory failure refractory to conventional support. This study aimed to determine whether extracorporeal membrane oxygenation (ECMO) is feasible in children with measles. A retrospective case series at a tertiary pediatric intensive care unit (PICU) with a regional ECMO program, of all children younger than 18 years with laboratory-confirmed measles who received ECMO during the August 2025–May 2026 outbreak. Of 49 children with measles admitted to the PICU, 10 received ECMO. Five were male; median age was 11 months (IQR, 10–24), and none had completed age-appropriate vaccination. Indications were septic shock or cardiorespiratory failure (*n* = 5), acute respiratory distress syndrome with septic shock (*n* = 2), hypoxemic respiratory failure (*n* = 2), and suspected myocarditis (*n* = 1). Seven received venoarterial, 1 veno-venoarterial, and 2 venovenous ECMO. Bacteremia preceded ECMO in 5 children and positive sputum culture in 8. *Candida tropicalis* candidemia developed in 3 of the first 5 patients, with none among the subsequent 5 after antifungal prophylaxis at cannulation. Left ventricular dysfunction was present in 7 of 9 assessed, 4 required decompression, and neuroimaging was abnormal in 4 of 8 imaged. Six children (60%) survived to discharge.

*Conclusions*: ECMO was feasible and potentially lifesaving in measles-associated cardiorespiratory failure, with survival comparable to other high-acuity pediatric indications, but the course was complicated by bacterial and fungal coinfection, cardiac dysfunction, and neurologic injury. These resource-intensive presentations occurred exclusively in unvaccinated children and were preventable, underscoring measles vaccination coverage as a public health priority.

**Table:** 

**What is Known:** • *Measles is resurgent worldwide and can cause severe respiratory, cardiac, and immune complications in children.* • *Measles-induced immune suppression predisposes to secondary bacterial and fungal infection.*	
**What is New:** • *First reported series of ECMO for measles: feasible, with 60% survival, but marked by coinfection, cardiac dysfunction, and neurologic injury.* • *Antifungal prophylaxis at ECMO cannulation was associated with elimination of candidemia in this cohort.*

**What is Known:**

• *Measles is resurgent worldwide and can cause severe respiratory, cardiac, and immune complications in children.*

• *Measles-induced immune suppression predisposes to secondary bacterial and fungal infection.*

**What is New:**

• *First reported series of ECMO for measles: feasible, with 60% survival, but marked by coinfection, cardiac dysfunction, and neurologic injury.*

• *Antifungal prophylaxis at ECMO cannulation was associated with elimination of candidemia in this cohort.*

## Introduction

Measles is among the most contagious of human pathogens, with a basic reproduction number estimated at 12 to 18, and despite the availability of a safe and effective vaccine for more than half a century, it remains an important cause of childhood death worldwide [1, 2]. After decades of decline, measles is resurgent. Since the COVID-19 pandemic, stagnating immunization coverage has been followed by a steep rise in disease, with an estimated 10.3 million cases and more than 107,000 deaths globally in 2023, most in children younger than 5 years [3]. High-income settings that had approached elimination have not been spared; in 2025, the USA recorded its highest case counts in more than 3 decades, most in undervaccinated communities [4]. This resurgence reflects vaccination gaps that widened during and after the pandemic, with the largest outbreaks concentrated where coverage has fallen below the threshold required for herd immunity [2, 4].

Measles concentrates in undervaccinated communities. The Jerusalem district has experienced recurrent outbreaks centered on communities with suboptimal vaccination coverage [5], where hospitalized children bear a heavy burden of complications [6]. Respiratory complications are the leading cause of measles death in young children [1], and their severity is compounded by measles-induced immune suppression. Measles virus infects and depletes memory lymphocytes, eliminating a large fraction of preexisting protective antibodies [7] and impairing reconstitution of the B-cell repertoire for months to years [8], thereby predisposing to secondary bacterial and fungal infection. Beyond pneumonia, severe measles may be complicated by laryngotracheobronchitis, acute encephalitis, and myocardial involvement, with case-fatality rising steeply in infants and in undervaccinated settings [1]. Most hospitalized children recover with supportive care, but a subset progress to critical illness, and a small number develop respiratory or circulatory failure that exhausts conventional support [6].

In a minority of children, measles precipitates cardiorespiratory failure, severe acute respiratory distress syndrome (ARDS), catecholamine-refractory septic shock, or myocardial dysfunction, that cannot be sustained by conventional intensive care. Extracorporeal membrane oxygenation (ECMO) is an established rescue therapy for refractory respiratory and cardiac failure in children, and its use has expanded across pediatric respiratory and cardiac indications. Yet the feasibility, complications, and survival of extracorporeal support specifically in measles have not been characterized, leaving clinicians without guidance when a child with measles deteriorates despite maximal conventional therapy. To our knowledge, no series has described ECMO in this setting.

During a large measles outbreak in Jerusalem in 2025–2026, an unprecedented number of children required ECMO for measles-associated cardiorespiratory failure. We describe their clinical characteristics, complications, and outcomes to inform the recognition and management of critically ill children with measles and to underscore the preventable nature of this disease. We also describe a practice change, introduction of antifungal prophylaxis at cannulation, that followed early observation of fungal coinfection.

## Methods

### Study design and participants

We conducted a retrospective case series at Hadassah University Medical Center, a tertiary pediatric referral center with a regional pediatric intensive care unit (PICU) and ECMO program in Jerusalem, Israel. We included all children (aged < 18 years) with laboratory-confirmed measles who received ECMO during the August 2025 to May 2026 measles outbreak. Measles was confirmed by real-time reverse-transcriptase polymerase chain reaction and/or detection of measles-specific IgM. This study followed the Strengthening the Reporting of Observational Studies in Epidemiology (STROBE) reporting guideline. The study was in accordance with the ethical standards of the responsible institutional committee on human experimentation and with the Helsinki Declaration of 1964, as revised in 2000. The Hadassah Medical Center Institutional Review Board approved the study (HMO-0460–12) and waived the requirement for informed consent given the retrospective design and use of deidentified data. Generative AI and AI-assisted technologies were not used.

### Data collection and definitions

We extracted demographic, clinical, laboratory, microbiological, echocardiographic, neuroimaging, and outcome data from the electronic medical record using a standardized form. Pre-ECMO values were those recorded closest to, and before, cannulation. The oxygenation index (OI) [9] and vasoactive-inotropic score (VIS) [10] were calculated according to standard definitions. ARDS was defined by the Pediatric Acute Lung Injury Consensus Conference (PALICC-2) criteria [9] and septic shock by the international consensus (Phoenix) criteria [11]. Bacteremia and candidemia were defined as growth of the organism from 1 or more peripheral blood cultures. Leukopenia was defined as a white blood cell count less than 5.0 × 10^3/μL and leukocytosis as greater than 17.0 × 10^3/μL. Left heart decompression denoted balloon atrial septostomy or surgical left-atrial venting during ECMO. Illness severity at cannulation was characterized by arterial blood gas values, the OI, and the VIS; microbiological data comprised blood, respiratory tract, and other cultures obtained before and during ECMO.

### ECMO and critical care management

ECMO was initiated for catecholamine-refractory cardiorespiratory failure or refractory hypoxemic respiratory failure unresponsive to conventional management, and was conducted according to institutional and Extracorporeal Life Support Organization (ELSO) protocols. The cannulation mode, venoarterial, veno-venoarterial, or venovenous, was selected on clinical grounds. Anticoagulation was achieved with unfractionated heparin, precannulation bolus of 50 units/kg followed by a continues infusion, starting at 20 units/kg/hour titrated to a target anti-factor Xa activity of 0.2–0.4 IU/mL, with dual monitoring by activated partial thromboplastin time targeted to 50–70 s; this reflected standard institutional practice for pediatric ECMO anticoagulation and was not altered a priori for children with measles. All children underwent serial echocardiography, and neuroimaging (cranial ultrasonography, computed tomography, or magnetic resonance imaging) was performed at the discretion of the treating team. Ventilator settings were adjusted to lung-protective targets after cannulation, and vasoactive support was weaned as native cardiac function recovered. After Candida tropicalis candidemia was identified in the earliest patients treated, empirical antifungal prophylaxis was introduced at the time of cannulation for all subsequent patients.

### Statistical analysis

Given the descriptive aim and small cohort, no formal hypothesis testing was undertaken. Continuous variables are summarized as median (interquartile range [IQR]) and categorical variables as number (percentage); denominators are stated where data were missing. Analyses were performed in Python version 3.12.3 (Python Software Foundation).

## Results

### Patient characteristics

**Fig. 1:**
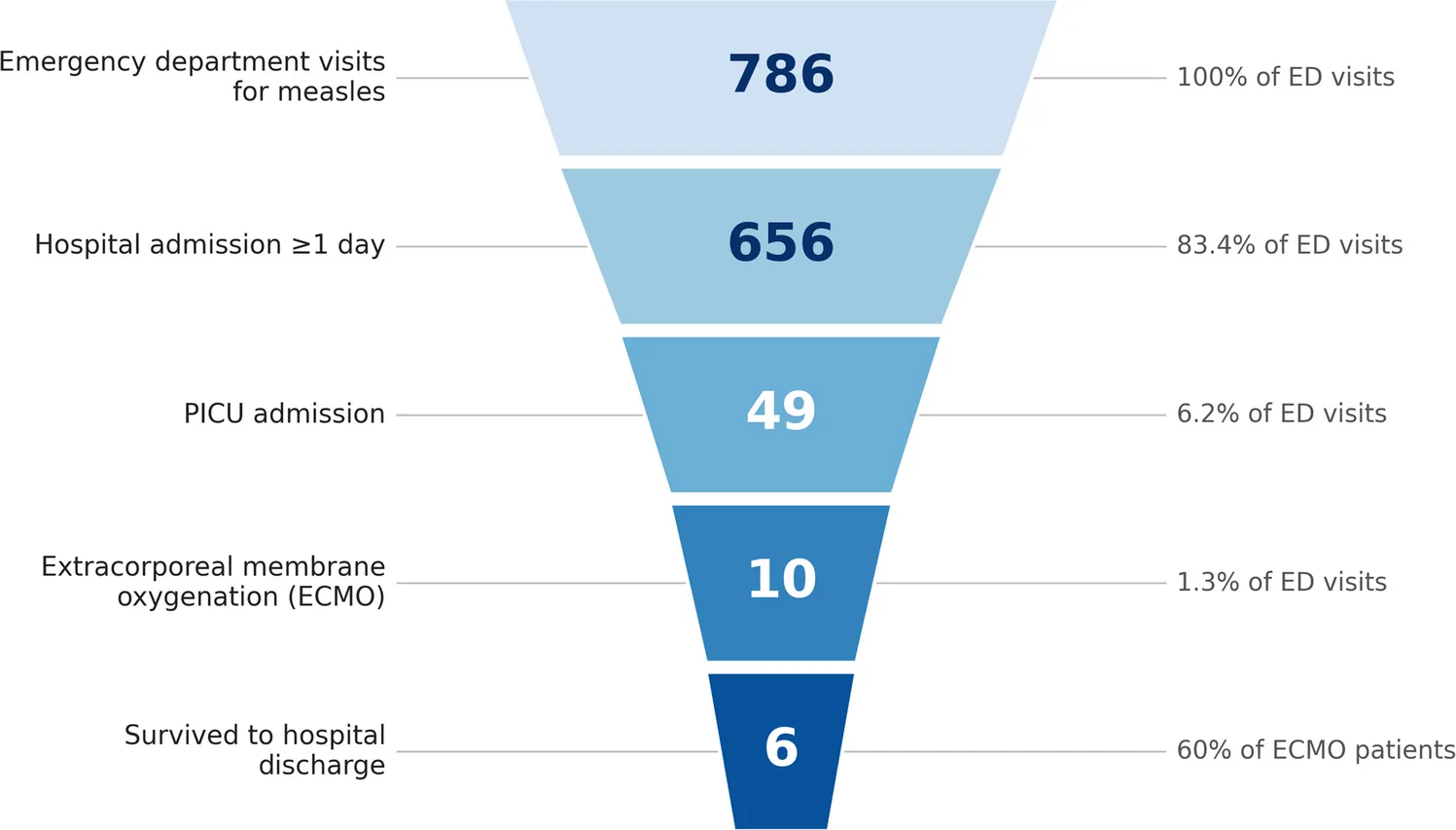
Severity and outcomes of measles among children during the 2025–2026 outbreak, Jerusalem, Israel. Legend: The funnel shows the number of children at each successive level of care. Among 786 children evaluated for measles in the emergency department (ED), 83.4% were admitted to the hospital for at least 1 day, 49 were admitted to the pediatric intensive care unit (PICU), and 10 received extracorporeal membrane oxygenation (ECMO); 6 of the 10 who received ECMO survived to hospital discharge. Percentages are relative to ED visits, except survival, which is relative to the 10 children who received ECMO

During the outbreak, 786 children were evaluated for measles in the pediatric emergency department, and 83.4% were admitted to the hospital for at least 1 day. Forty-nine children were admitted to the PICU, of whom 10 received ECMO and constituted the study cohort (Fig. 1).

Five were male; median age was 11 months (IQR, 10–24) and median weight was 9.2 kg (IQR, 8.0–11.5); 7 of 10 children were younger than 15 months. None had completed age-appropriate measles vaccination. All 10 received systemic corticosteroids and 9 received inhaled nitric oxide. Baseline characteristics are summarized in Table 1.

**Table 1:** Baseline characteristics, illness severity, and laboratory findings

Characteristic	Value (*N* = 10)
Demographics and presentation	
Age, median (IQR), mo	11 (10–24)
Male sex, No	5
Weight, median (IQR), kg	9.2 (8.0–11.5)
Completed age-appropriate measles vaccination, No	0
Systemic corticosteroids, No	10
Inhaled nitric oxide, No	9
Indication for ECMO, No	
Septic shock or cardiorespiratory failure	5
ARDS with septic shock	2
Hypoxemic respiratory failure	2
Suspected myocarditis	1
Severity at cannulation, median (IQR)	
pH	7.15 (7.06–7.20)
Lactate, mg/dL [mmol/L]	38.7 (20.7–63.1) [4.3 (2.3–7.0)]
Oxygenation index	28 (24–29)
Vasoactive-inotropic score	35 (17–52)
Fraction of inspired oxygen, %	100 (89–100)
Partial pressure of carbon dioxide, mm Hg	52 (50–55)
Partial pressure of oxygen, mm Hg	57 (55–62)
Bicarbonate, mEq/L	19.3 (15.2–25.8)
Laboratory findings at cannulation, median (IQR)	
White blood cells, × 103/μL	2.4 (1.0–3.3)
Hemoglobin, g/dL	10.2 (9.1–11.4)
Platelets, × 103/μL	184 (56–270)
C-reactive protein, mg/dL	12.0 (8.5–14.7)
Aspartate aminotransferase, U/L	240 (180–355)
International normalized ratio	1.50 (1.11–1.73)
Fibrinogen, mg/dL	205 (130–342)
**Cardiac function**	
LV or biventricular dysfunction, No./assessed	7/9
Severe, No	5

*ARDS* acute respiratory distress syndrome, *ECMO* extracorporeal membrane oxygenation, *IQR* interquartile range, *LV* left ventricular. Echocardiography was not recorded for 1 patient

### Illness severity and laboratory findings

ECMO was initiated for septic shock or cardiorespiratory failure in 5 children, ARDS with septic shock in 2, hypoxemic respiratory failure in 2, and suspected myocarditis in 1; a septic-shock component was present in 7. At cannulation, children were critically ill, with severe metabolic and respiratory derangement (median pH, 7.15 [IQR, 7.06–7.20]; lactate, 38.7 mg/dL [4.3 mmol/L] [IQR, 20.7–63.1 mg/dL; 2.3–7.0 mmol/L]; OI, 28 [IQR, 24–29]; and VIS, 35 [IQR, 17–52]), and all required high-concentration oxygen and vasoactive support. Hematologic findings reflected the immune suppression of measles: the median white blood cell count was 2.4 × 10^3/μL (IQR, 1.0–3.3), with leukopenia in 8 children and leukocytosis in 1, and the median platelet count was 184 × 10^3/μL (IQR, 56–270). Inflammatory and hepatic markers were elevated (median C-reactive protein, 12.0 mg/dL [120 mg/L] [IQR, 8.5–14.7 mg/dL; 85–147 mg/L]; median aspartate aminotransferase, 240 U/L [IQR, 180–355]), and coagulopathy was common (median international normalized ratio, 1.50 [IQR, 1.11–1.73]; median fibrinogen, 205 mg/dL [2.05 g/L] [IQR, 130–342 mg/dL; 1.3–3.42 g/L]). Gas exchange was severely impaired despite a median fraction of inspired oxygen of 100% (median partial pressure of carbon dioxide, 52 mm Hg; partial pressure of oxygen, 57 mm Hg; bicarbonate, 19.3 mEq/L). Echocardiography, available in 9 children, showed left ventricular or biventricular dysfunction in 7, severe in 5 (Table 1).

### ECMO support

Seven children received venoarterial, 1 veno-venoarterial, and 2 venovenous ECMO; 1 venoarterial run was converted from peripheral neck to central cannulation. The median duration of ECMO was 10.5 days (IQR, 6.0–14.2; range, 2–60). The predominance of venoarterial configurations reflected shock and cardiac dysfunction as the leading indications.

### Microbiology

Bacteremia preceded ECMO in 5 children (*Streptococcus pneumoniae*, 2; nontypeable *Haemophilus influenzae*, 2; and group A streptococcus, 1). Positive sputum culture preceded ECMO in 8 children (*Staphylococcus aureus*, 7; group A streptococcus, 2; and *Klebsiella pneumoniae*, 1). Candidemia, all due to *Candida tropicalis*, developed during ECMO in 3 children, all among the first 5 patients treated; whole-genome sequencing of the isolates excluded a common-source outbreak. After antifungal prophylaxis was introduced at cannulation, none of the subsequent 5 children developed candidemia (Table 2).

**Table 2:** ECMO support, microbiology, complications, and outcomes

Characteristic	Value (*N* = 10)
ECMO support	
Venoarterial, No	7
Veno-venoarterial, No	1
Venovenous, No	2
ECMO duration, median (IQR) [range], d	10.5 (6.0–14.2) [2–60]
Infection	
Pre-ECMO bacteremia, No	5
* S. pneumoniae*/nontypeable *H. influenzae*/group A strep	2/2/1
Positive pre-ECMO sputum culture, No	8
* S. aureus*/group A strep/*K. pneumoniae*	7/2/1
On-ECMO candidemia (*C. tropicalis*), No	3
First 5 patients/subsequent 5 patients	3/0
Complications	
Left heart decompression, No	4
Balloon atrial septostomy/surgical LA vent	3/1
Abnormal neuroimaging, No./imaged	4/8
Acute kidney injury, No	10
Hemofiltration, No	9
Abnormal liver enzymes, No	7
Bleeding, No	3
Outcome	
Survival to hospital discharge, No. (%)	6 (60)
Hypoxemic respiratory failure	2/2
Septic shock or cardiorespiratory	4/7
Suspected myocarditis	0/1

*ECMO* extracorporeal membrane oxygenation, *LA* left atrium. All candidemia was due to *Candida tropicalis*, occurring only among the first 5 patients treated, before antifungal prophylaxis was introduced at cannulation

### Complications and outcomes

Severe left ventricular dysfunction necessitated left heart decompression in 4 children (balloon atrial septostomy, 3; surgical left-atrial venting, 1), of whom 1 survived. Neuroimaging was performed in 8 children and was abnormal in 4, comprising subdural hematoma, multiple cerebral infarcts, deep gray-matter diffusion restriction, and global cerebral edema with herniation (1 each); 1 of these 4 children survived. Acute kidney injury occurred in all 10 children, and 9 required hemofiltration via the ECMO circuit; abnormal liver enzymes were present in 7 children, and clinically significant bleeding occurred in 3. In 2 of them, anticoagulation was subsequently modified from the protocol target because of clinically significant bleeding: heparin was discontinued in 1 child after mucosal bleeding developed, and withheld entirely in a second child with combined mucosal and pulmonary bleeding. Six children (60%) survived to hospital discharge. Median PICU length of stay was 32.5 days (IQR, 20.2–51.2; range, 3–75) and was shorter among children who died (16.5 days [IQR, 9.8–31.0]) than among survivors (39.5 days [IQR, 32.2–51.2]), consistent with early mortality in the most severe presentations. Outcomes varied by indication: both children supported for hypoxemic respiratory failure survived (2 of 2), as did 4 of 7 with a septic-shock indication, whereas the child with suspected myocarditis died. A comparison of demographic, laboratory, and complication data between children who died and those who survived, including a markedly higher rate of bleeding among those who died (3 of 4 vs 0 of 6), is presented in Table 3. Individual patient-level data are shown in Table 4.

**Table 3:** Comparison of clinical, laboratory, and outcome parameters between children who died and children who survived to hospital discharge

Parameter	Died (*n* = 4)	Survived (*n* = 6)
Demographics		
Age, median (IQR), mo	20 (11–28)	10 (8–13)
Male sex, No. (%)	1 (25)	4 (67)
Weight, median (IQR), kg	11.0 (9.2–12.0)	8.2 (8.0–9.6)
ECMO characteristics		
Configuration, No	VA 3, VVA 1	VA 4, VV 2
ECMO duration, median (IQR), d	16 (10–31)	8 (6–11)
Illness severity at cannulation, median (IQR)		
pH	7.12 (7.04–7.18)	7.16 (7.10–7.28)
Lactate, mmol/L	10.8 (4.3–16.9)	3.0 (1.4–6.0)
Oxygenation index	28 (27–42)	28 (20–29)
Vasoactive-inotropic score	51 (50–59)	18 (15–20)
Fraction of inspired oxygen, %	100 (100–100)	92 (81–100)
Partial pressure of CO2, mm Hg	50 (49–51)	54 (52–64)
Partial pressure of O2, mm Hg	56 (53–63)	57 (55–62)
Bicarbonate, mEq/L	14.9 (14.1–15.7)	25.8 (21.5–26.9)
Laboratory findings at cannulation, median (IQR)		
White blood cells, × 103/μL	1.7 (0.9–2.7)	2.7 (1.6–5.5)
Hemoglobin, g/dL	12.3 (10.5–14.2)	9.4 (8.8–10.5)
Platelets, × 103/μL	94 (27–192)	220 (126–270)
C-reactive protein, mg/dL	8.8 (7.4–11.4)	13.1 (11.6–14.7)
Creatinine, mg/dL	0.60 (0.39–0.82)	0.39 (0.28–0.94)
ALT, U/L	92 (31–162)	52 (43–70)
AST, U/L	231 (203–418)	242 (146–355)
International normalized ratio	2.08 (1.63–2.50)	1.12 (1.05–1.48)
Fibrinogen, mg/dL	100 (74–133)	312 (239–456)
Lymphocytes, × 103/μL	1.40 (0.65–2.03)	1.30 (0.75–1.81)
Neutrophils, × 103/μL	0.15 (0.08–0.42)	1.10 (0.55–3.38)
Albumin, g/dL	1.9 (1.7–2.1)	3.2 (2.5–3.6)
Laboratory findings at PICU admission, median (IQR)		
White blood cells, × 103/μL	1.7 (0.9–2.7)	2.7 (1.6–4.1)
Absolute lymphocyte count, × 103/μL	1.4 (0.7–2.0)	0.8 (0.7–1.6)
Absolute neutrophil count, × 103/μL	0.2 (0.1–0.4)	1.1 (0.6–2.8)
Complications, No. (%)		
Acute kidney injury	4 (100)	6 (100)
Continuous renal replacement therapy	4 (100)	5 (83)
Abnormal liver function tests	4 (100)	3 (50)
Bleeding	3 (75)	0 (0)
**Outcome**		
PICU length of stay, median (IQR), d	16.5 (9.8–31.0)	39.5 (32.2–51.2)

*ALT* alanine aminotransferase, *AST* aspartate aminotransferase, *ECMO* extracorporeal membrane oxygenation, *IQR* interquartile range, *PICU* pediatric intensive care unit, *VA* venoarterial, *VV* venovenous, *VVA* veno-venoarterial. Given the small sample size (4 deaths, 6 survivors), no formal statistical comparison (e.g., hypothesis testing) was performed; values are presented descriptively and should be interpreted as exploratory, not as evidence of a significant association with outcome

**Table 4:** Individual patient-level characteristics and outcomes

Pt	Age, mo	Sex	Indication	ECMO mode	ECMO run (days)	Pre-ECMO bacteremia	On-ECMO candidemia	Echocardiography	Neuroimaging	LHD	PICU LOS (days)	Outcome
1	28	M	Septic shock	VA (neck)	21	*S. pneumoniae*	None	Severe LV + moderate RV	Subdural hematoma	BAS	21	Died
2	11	F	Suspected myocarditis	VA neck to central	60	None	*C. tropicalis* (d54)	Severe biventricular	Multiple infarcts	Surgical LA vent	61	Died
3	11	M	Septic shock	VA (neck)	6	*H. influenzae* (NT)	None	Severe biventricular	DWI restriction, capsule/BG	—	46	Survived
4	10	M	Hypoxemic resp failure	VV	4	*H. influenzae* (NT)	*C. tropicalis* (d3)	Mildly reduced LV	Not done	—	53	Survived
5	29	F	Septic shock	VA (neck)	12	None	*C. tropicalis* (d5)	Severe LV	Global edema, herniation	—	12	Died
6	39	F	Septic shock	VA (neck)	6	None	None	Severe LV	Normal (MRI)	BAS	33	Survived
7	11	F	Septic shock	VVA	2	Group A strep	None	LV dysfunction	Not done	BAS	3	Died
8	14	M	Hypoxemic resp failure	VV	10	None	None	Normal LV	Normal (US)	—	20	Survived
9	6	M	ARDS + septic shock	VA (neck)	11	None	None	Normal biventricular	Normal (US)	—	75	Survived
10	8	F	ARDS + septic shock	VA (neck)	15	*S. pneumoniae*	None	Not recorded	Normal (US)	—	32	Survived

*ARDS* acute respiratory distress syndrome, *BAS* balloon atrial septostomy, *BG* basal ganglia, *DWI* diffusion-weighted imaging, *LA* left atrial, *LHD* left heart decompression, *PICU LOS* pediatric intensive care unit length of stay, *LV* left ventricular, *MRI* magnetic resonance imaging, *NT* nontypeable, *Pt* patient, *RV* right ventricular, *US* ultrasonography, *VA* venoarterial, *VV* venovenous, *VVA* veno-venoarterial. A dash (—) indicates that left heart decompression was not performed. Echocardiography was not recorded for patient 10

## Discussion

During a single measles outbreak, 10 children, most of them infants, required ECMO for measles-associated cardiorespiratory failure, and 6 (60%) survived to hospital discharge. To our knowledge, this is the first reported series of ECMO for measles. The cohort was characterized by a heavy burden of bacterial and fungal coinfection, frequent left ventricular dysfunction requiring decompression, and a substantial rate of neurologic injury, situating these intensive-care findings within a broader failure of measles prevention.

The microbiological profile reflects the profound immune suppression that follows measles, which depletes memory lymphocytes and preexisting antibodies for months to years [7, 8]. Half of our patients had bacteremia before cannulation, and most had positive respiratory cultures, mirroring the complication profile of hospitalized children in prior Jerusalem outbreaks [6]. The organisms recovered, pneumococcus, *Haemophilus influenzae*, group A streptococcus, and *Staphylococcus aureus*, are pathogens classically responsible for post-measles bacterial superinfection, underscoring the vulnerability created by transient immune paralysis. More striking was *Candida tropicalis* candidemia in 3 of the first 5 patients; whole-genome sequencing excluded a common-source outbreak, implicating host susceptibility rather than nosocomial transmission. Although a before-and-after observation cannot establish efficacy, the absence of candidemia in the subsequent 5 children after antifungal prophylaxis was introduced at cannulation is biologically plausible in the setting of measles-induced immunoparesis and warrants prospective evaluation. Invasive candidiasis is a recognized and frequently fatal complication in immunocompromised and extracorporeally supported children, and *Candida tropicalis* is among the more virulent non-albicans species; its clustering exclusively in our earliest, unprophylaxed patients is consistent with a window of maximal measles-induced immunoparesis. In the interim, a low threshold for antifungal coverage seems prudent in children requiring ECMO for measles.

Cardiac involvement was prominent and, in our experience, underrecognized as a feature of severe measles. Severe left ventricular or biventricular dysfunction was present in most children, and 4 required left heart decompression. Myocardial dysfunction likely reflects both sepsis-associated cardiomyopathy and direct viral myocarditis, a rare but recognized complication of measles; [1] although ECMO supports children with myocarditis with a reported survival of approximately 61% [12], the single child supported for suspected myocarditis in our series died. Left ventricular distension is an inherent hazard of venoarterial ECMO, in which retrograde aortic flow increases afterload on the failing ventricle and can precipitate pulmonary edema and hemorrhage unless the left heart is decompressed [13]. The high decompression rate we observed, and the poor survival of these children, supports early echocardiographic surveillance with a low threshold for venting.

Neurologic injury was frequent and carried a poor prognosis; 4 of 8 imaged children had abnormal findings, ranging from subdural hematoma and cerebral infarction to global cerebral edema with herniation, and only 1 survived. This rate greatly exceeds the background frequency of acute neurologic complications reported to the ELSO registry for children on ECMO [14], likely reflecting the convergence of measles-associated encephalitis, hypoxic-ischemic injury from refractory shock, and the hemorrhagic and thrombotic risks of extracorporeal support, which cannot be disentangled in a retrospective cohort, particularly because lumbar puncture is rarely feasible during anticoagulated ECMO. Measles itself causes a spectrum of central nervous system disease, from acute postinfectious encephalitis to, rarely, later subacute sclerosing panencephalitis [1], and the acute injuries we observed may compound this intrinsic neurotropism with the circulatory and hematologic stresses of critical illness and extracorporeal support. The prognostic weight of these findings argues for systematic neuromonitoring and neuroimaging in this population.

Renal and hepatic involvement were near-universal: all 10 children met criteria for acute kidney injury and 9 required hemofiltration via the ECMO circuit, and abnormal liver enzymes were present in 7. Bleeding occurred in 3 children, all of whom died, and anticoagulation was modified or withheld in response in 2 of these cases, a pattern consistent with bleeding as a marker of, or contributor to, the most severe courses rather than a routine complication of ECMO itself. Children who died had a shorter PICU length of stay than survivors, reflecting early mortality in the most severe presentations rather than a more protracted course.

Despite this severity, 60% of children survived, consistent with reported outcomes for ECMO in pediatric sepsis and septic shock, in which pooled survival approaches 60% [15], and with cumulative registry survival across pediatric ECMO indications [16]. Measles, though not separately cataloged among ECMO indications, should be regarded as a legitimate indication when conventional management fails. Notably, the 2 children supported for primary hypoxemic respiratory failure both survived, consistent with the more favorable outcomes of pediatric respiratory ECMO, whereas the combination of septic shock, left ventricular dysfunction requiring decompression, and neurologic injury identified a higher-risk phenotype. Overall survival of 60% is consistent with contemporary pediatric ECMO experience across indications, indicating that measles per se does not portend a futile course.

Above all, these findings carry a clear public health message. Every child in this series developed life-threatening, resource-intensive disease from a vaccine-preventable infection, and most were infants too young for routine vaccination who depend on community immunity for protection [17]. Sustained vaccination coverage of at least 95% is required to interrupt transmission and protect this youngest group [2, 18], and adjunctive vitamin A, recommended by the World Health Organization for all children with acute measles, reduces mortality in children younger than 2 years [19], the age group that comprised most of our cohort. The immune amnesia induced by measles further erodes population-level protection against unrelated pathogens, amplifying the downstream morbidity of every outbreak [7, 8], and coverage has fallen below the elimination threshold in many communities, including the undervaccinated populations in which recent Jerusalem outbreaks have clustered [5]. None of these measures substitutes for the primary prevention afforded by timely 2-dose immunization. Because infants younger than the age of first vaccination cannot yet be directly protected, their safety depends on interrupting community transmission; maternal immunization and timely, high childhood coverage are complementary strategies to close this window.

The resource intensity of these cases, prolonged ECMO, multidisciplinary critical care, and management of coinfection and neurologic injury, has direct implications for health systems facing measles resurgence and argues for anticipatory planning of pediatric ECMO capacity during outbreaks. Prospective, multicenter collaboration, including systematic capture of measles among ECMO indications in international registries, would help define the role, optimal timing, and outcomes of extracorporeal support in this population and test whether antifungal prophylaxis and early left heart decompression improve survival. Until such data accrue, our experience supports offering ECMO to children with reversible, measles-associated cardiorespiratory failure at experienced centers, while recognizing that the greatest gains lie upstream, in prevention. For the practicing intensivist, the immediate implications are to anticipate coinfection and cardiac and neurologic complications, to involve infectious diseases and cardiology specialists early, and to counsel families that the underlying illness was preventable.

### Limitations

This study has limitations. It is a single-center, retrospective case series without a control group; the small sample precludes formal statistical inference, and patient selection for ECMO was not protocolized. Microbiological yield may underestimate the true infection burden, and the apparent benefit of antifungal prophylaxis derives from an uncontrolled before-and-after comparison requiring prospective confirmation. As a descriptive series, it cannot establish causal relationships, and the single-outbreak, single-center design limits external validity.

## Conclusions

In this case series, measles caused cardiorespiratory failure severe enough to require ECMO in previously healthy children, and ECMO was feasible and potentially lifesaving. The burden of bacterial and fungal coinfection, left ventricular dysfunction, and neurologic injury defines the particular challenges of supporting these children. Most importantly, every case was a preventable consequence of inadequate vaccination coverage: measles is a vaccine-preventable disease, and protecting the youngest children depends on sustained, high community immunity achieved through effective immunization.

## Data Availability

The datasets generated and/or analyzed during the current study are available from the corresponding author on reasonable request.

## Abbreviations

AKI: Acute kidney injury
ARDS: Acute respiratory distress syndrome
COVID-19: Coronavirus disease 2019
ECMO: Extracorporeal membrane oxygenation
ELSO: Extracorporeal Life Support Organization
IgM: Immunoglobulin M
IQR: Interquartile range
OI: Oxygenation index
PALICC-2: Second Pediatric Acute Lung Injury Consensus Conference
PICU: Pediatric intensive care unit
STROBE: Strengthening the Reporting of Observational Studies in Epidemiology
VIS: Vasoactive-inotropic score
